# Comparative hologenomics of two *Ixodes scapularis* tick populations in New Jersey

**DOI:** 10.7717/peerj.12313

**Published:** 2021-11-09

**Authors:** Dana C. Price, Reilly N. Brennan, Nicole E. Wagner, Andrea M. Egizi

**Affiliations:** 1 Department of Entomology, Center for Vector Biology, Rutgers, The State University of New Jersey, New Brunswick, NJ, United States of America; 2 Tick-Borne Disease Laboratory, Monmouth County Mosquito Control Division, Tinton Falls, NJ, United States of America

**Keywords:** Blacklegged tick, *Ixodes scapularis*, Deer tick, *Rickettsia buchneri*, Hologenomics, Metagenomics, Vector biology, Holobiont

## Abstract

Tick-borne diseases, such as those transmitted by the blacklegged tick *Ixodes scapularis*, are a significant and growing public health problem in the US. There is mounting evidence that co-occurring non-pathogenic microbes can also impact tick-borne disease transmission. Shotgun metagenome sequencing enables sampling of the complete tick hologenome—the collective genomes of the tick and all of the microbial species contained therein, whether pathogenic, commensal or symbiotic. This approach simultaneously uncovers taxonomic composition and allows the detection of intraspecific genetic variation, making it a useful tool to compare spatial differences across tick populations. We evaluated this approach by comparing hologenome data from two tick samples (*N* = 6 ticks per location) collected at a relatively fine spatial scale, approximately 23 km apart, within a single US county. Several intriguing variants in the data between the two sites were detected, including polymorphisms in both in the tick’s own mitochondrial DNA and that of a rickettsial endosymbiont. The two samples were broadly similar in terms of the microbial species present, including multiple known tick-borne pathogens (*Borrelia burgdorferi*, *Babesia microti,* and *Anaplasma phagocytophilum*), filarial nematodes, and *Wolbachia* and *Babesia* species. We assembled the complete genome of the rickettsial endosymbiont (most likely *Rickettsia buchneri*) from both populations. Our results provide further evidence for the use of shotgun metagenome sequencing as a tool to compare tick hologenomes and differentiate tick populations across localized spatial scales.

## Introduction

The black-legged tick, *Ixodes scapularis* (Say) is responsible for the greatest volume of vector-borne disease cases in the United States. Lyme disease, caused by the spirochete *Borrelia burgdorferi* and transmitted by *I. scapularis,* is estimated to afflict over 300,000 people annually, primarily in the US northeast and Midwest (Hinckley et al., 2014; Nelson et al., 2015). In addition to significant impacts on patients’ quality of life, Lyme disease is estimated to cost as much as $1.3 US billion per year to diagnose and treat (Adrion et al., 2015). *Ixodes scapularis* is also the vector of several additional human pathogens, including *Anaplasma phagocytophilum*, *Babesia microti*, *Borrelia miyamotoi*, and Powassan encephalitis virus (Nelder et al., 2016).

In New Jersey, the first Lyme disease cases were described in the late 1970s (Slade & Lenz, 1982; Ward, 1981) and it quickly became endemic in the state. The distribution was primarily limited to the southern two-thirds of the state until the late 1980s, coinciding with a northward expansion of the vector (Schulze, Jordan & Hung, 1998). Monmouth County has long been endemic for *I. scapularis* and *B. burgdorferi* and the county was home to a focus of >50% of the state’s cases between 1978 and 1982 (Bowen et al., 1984). With its long tradition of specialized research including pathogen surveys and studies on vector and host ecology (*e.g.*, Schulze et al., 2013; Schulze et al., 2006) Monmouth is an ideal context in which to study *I. scapularis* population dynamics.

Historically, the majority of microbial surveys in ticks have focused on pathogen detection. In recent years, however, there has been a growing awareness that non-pathogenic and commensal microbes can affect both host fitness and pathogen transmission in important ways (Bonnet et al., 2017). For example, the rickettsial endosymbionts of *I. scapularis* and the western black-legged tick *I. pacificus* encode a complete folate biosynthesis pathway that is absent in the ticks, and may supplement a hematophagous lifestyle (Hunter et al., 2015). As the holobiont encompasses the full range of microbial species living associated with a host, as well as the host itself, the hologenome thus comprises the sum of the host genome and all associated microbial genomes (Simon et al., 2019). Comparisons of hologenomes across tick species have revealed coevolutionary patterns between ticks and endosymbionts (Díaz-Sánchez et al., 2019) and underlined the utility of studying these interdependent taxa in concert. However, it is possible that within-species comparisons of hologenomes may also provide important insights, such as relative spatial scales of tick *vs.* tick-borne pathogen movement and genetic divergence.

Research that synthesizes vector host genetics, population structure, microbiome composition, and vectorial capacity may provide further understanding of factors governing pathogen transmission (Griffiths et al., 2018). Such studies are lacking with regard to ticks despite their prominent status as an arthropod vector. To address this, Lado et al. (2020) recently sampled a broad selection of individual North American *Dermacentor variabilis* and used ddRAD-seq SNP markers (Lado et al., 2019) in conjunction with 16S microbial amplicon sequencing to describe a tick microbiome highly dominated by few taxa and varying by geographic region. Low levels of host genetic diversity made integration of these datasets and further inference of population variation and structure difficult, however.

Shotgun metagenomics is a common-place practice of sequencing the total DNA present in a sample, without prior PCR amplification targeting a particular region or taxon (Miller et al., 2013), which can introduce bias into the results. This offers a unique opportunity to simultaneously detect the complete suite of pathogenic, non-pathogenic and symbiotic microbes present in tick tissues, while also affording phylogenetic comparisons with related species and distinction of unique strains at loci beyond those used commonly for amplicon sequencing.

We utilized shotgun sequencing to characterize and compare hologenome data from two pools of *I. scapularis* females collected at two sites with abundant populations within Monmouth County, NJ to determine if there were observable differences (inclusive of the host and microbial constituent genomes) at this spatial scale. To corroborate our taxonomic identifications, we also applied a typical amplicon sequencing approach (16S small subunit ribosomal rDNA) to the same samples. Our goal was not a pathogen survey *per se* as only six ticks from each site were used, but rather to assess any divergence within the tick hologenome between these two sites and to evaluate the feasibility of similar methodologies for future use in more comprehensive studies of localized movements of ticks and their associated pathogens.

## Materials & Methods

### Tick collection

Two sites in Monmouth County, New Jersey were chosen for tick collection, occurring in geologically distinct regions of the county (inner *vs.* outer coastal plain deposits) (Schulze et al., 2011). Naval Weapons Station Earle (NWSE) is a 41-km^2^ secured military facility located in the outer coastal plain in a mixed hardwood and pine forest. Perrineville Lake Park (PVIL) is a ∼5 km^2^ public park located in the inner coastal plain with primarily hardwood deciduous forest. According to historical data, PVIL was colonized by *I. scapularis* slightly later than NWSE, occurring sometime between 1981 and 1985 (Schulze et al., 1986). The two sites are approximately 23 km (14.5 mi) apart.

Ticks were collected by dragging a 1-m^2^ sailcloth drag across plots at each site (reference site lat/lon: NWSE: 40.2705, −74.1633; PVIL: 40.2408, −74.4313), known to be productive locations for *I. scapularis* (Dolan et al., 2009; Schulze et al., 2013), during November 20–21, 2017. Female *I. scapularis* ticks were removed from the drags with forceps and placed in glass vials with a small damp paper towel to be kept alive until dissection. Species identification and sex was further confirmed using established keys (Keirans & Litwak, 1989).

### DNA extraction

Six live *I. scapularis* females from each site (total across both sites *N* = 12) were removed from the vials, surface sterilized in 1% sodium hypochlorite (bleach) for 2 min, and washed twice in sterile water. Ticks were embedded in warm paraffin, and an incision was made around the edge of the dorsum using a microscalpel. The dorsal cuticle and scutum were removed and the salivary glands, midgut, rectal sac and ovaries were excised as one unit and placed in AllProtect Tissue Reagent (Qiagen Inc., Valencia, CA). After the dissections were complete, the excised tissue samples from each site were removed from AllProtect and pooled in 2 ml microfuge tubes, where 350 µl of buffer RLT Plus (Qiagen Inc., Valencia, CA) was added along with a five mm stainless steel bead. Samples were homogenized in a Qiagen TissueLyser for 2 min at 30 Hz. The remainder of the extraction protocol was carried out per the AllPrep DNA/RNA Mini handbook (Qiagen Inc., Valencia, CA) and eluted in 50 µl of RNAse-free water.

### Shotgun metagenome sequencing

Approximately 50 ng of extracted DNA was used to construct an Illumina sequencing library for each sample using the Nextera FLEX library prep kit (Illumina Inc., San Diego, CA) following manufacturer’s protocol. Both libraries were multiplexed on an Illumina MiSeq instrument and sequenced on a single run in 305 bp × 305 bp paired-end mode using a 600-cycle MiSeq Reagent Kit v3 (Illumina Inc., San Diego, CA, USA).

Raw reads were adapter and quality trimmed using bbduk (part of the bbmap package; https://sourceforge.net/projects/bbmap/) using parameters “minlen = 50 qtrim = rl trimq = 15 ktrim = r *k* = 20 mink = 8 hdist = 1”. Trimmed data were assembled to metagenome scaffolds using SPAdes 3.12.0 (Bankevich et al., 2012) with a *k*-mer range of (33, 55, 77, 99 and 127). Homology searches using the raw reads were performed using both the DIAMOND protein sequence aligner (Buchfink, Xie & Huson, 2015) against the NCBI ‘nr’ database (downloaded 10/26/2019) with an e-value threshold of 1 × 10^−5^ and additionally against the NCBI ‘nt’ nucleotide database with the addition of two *I. scapularis* genome assemblies retrieved from VectorBase (Giraldo-Calderón et al., 2015) (strains ISE6 (Miller et al., 2018)) and Wikel (Gulia-Nuss et al., 2016) using BLASTn v.2.9.0 (MEGABLAST algorithm; Altschul et al., 1990) on the Rutgers Office of Advanced Research Computing amarel cluster. The NCBI taxonomic string for each DIAMOND or BLAST hit accession was retrieved using the taxonomizr R package (https://cran.r-project.org/web/packages/taxonomizr/) and the corresponding annotation was performed with the Biopython SeqIO module (https://biopython.org/).

### 16S amplicon sequencing

To rule out the possibility that a disproportionate abundance of tick DNA affected the detection of microbial DNA in the shotgun results, a 16S SSU rRNA amplicon library was also prepared for each sample using 1 µL of DNA extraction and the primers and protocol of the Illumina 16S Metagenomic Sequencing Library Preparation manual (available from https://support.illumina.com/). The protocol targets the ca. 402 bp 16S hypervariable regions V3–V4 using the 0341 and 0785 primers developed by Herlemann et al. (2011) modified to include Illumina adapter overhangs:

F overhang: 5′-TCGTCGGCAGCGTCAGATGTGTATAAGAGACAG-[F primer]

R overhang: 5′-GTCTCGTGGGCTCGGAGATGTGTATAAGAGACAG-[R primer].

Each amplicon library was sequenced on an Illumina MiSeq in 305 bp × 305 bp paired end mode using approximately 1% of a 600-cycle MiSeq Reagent Kit v3.

The 16S SSU paired amplicon reads were quality trimmed and merged to a single fragment using bbmerge (https://sourceforge.net/projects/bbmap) with parameters “qtrim = r trimq = 15 loose = t”. Merged amplicons were queried against the NCBI ‘nt’ database using BLASTn (*e*-value = 1 × 10^−20^), and NCBI taxonomic nomenclature was assigned using taxonomizr as above.

### Variant detection

To identify single nucleotide variants (SNVs) in the host tick and *Rickettsia* endosymbiont genomes, we separately mapped short reads from both locations (NWSE and PVIL) to the *I. scapularis* ISE6 reference genomic DNA sequence (NCBI accession GCF_002892825.2; initiated from ticks collected in GA, USA) and to the *Rickettsia* endosymbiont of *Ixodes scapularis* str. Wikel genome sequence + 3 associated plasmids (NCBI BioProject PRJNA33979; initiated from ticks collected in CT, USA). This was performed using bbmap (https://sourceforge.net/projects/bbmap/) with a minimum read sequence identity threshold of 90% (minid = 0.90) and visualized using Geneious Prime 2020 (http://www.geneious.com). Single nucleotide variants were annotated using the Geneious variant detection tool (minimum coverage = 10×, minimum variant frequency = 70% (defined as number of reads containing the variant divided by number of reads mapped at each site)). We present only polymorphisms present in the short-read data between our two samples and not those present between either sample and the reference (tick or symbiont) genome. Significance (associated *p*-value) of each SNV was defined as the probability of encountering each variant by chance due to sequencing error after averaging base quality scores for all nucleotides that comprise the SNV and reference separately (as implemented in Geneious Prime 2020).

As we were unable to identify a public complete mitochondrial genome sequence for *I. scapularis*, we first mapped both short-read libraries concurrently to the published mitochondrial genome of the closest sister taxon, *I. ricinus* (NCBI accession NC_018369) as above using bbmap with a nucleotide similarity of 85%. This mapping spanned the entire length of the *I. ricinus* reference with an average coverage of >600x. A majority-rule (50%) consensus sequence was then generated using this mapping that represented our *I. scapularis* mitochondrial genome reference and annotated with MITOS (Bernt et al., 2013). Each library was then separately mapped to this reference, and single nucleotide variants (SNVs) were called using Geneious (minimum coverage = 100x, minimum variant frequency = 25%).

### Phylogenetic analysis

The *Wolbachia* phylogeny was constructed by first querying the NCBI ‘nr’ protein database *via* BLASTx with assembled genome scaffold NODE_632479, which was identified as being of *Wolbachia* provenance (below). All available hits were retrieved (*n* = 44) and these proteins were aligned with the ORF encoded on our query scaffold using MAFFT v.7.2 (Katoh & Standley, 2013). This alignment was used to construct a maximum-likelihood phylogenetic tree using IQTREE v1.6 (Nguyen et al., 2015) with automatic model selection and nodal support values calculated using 2,000 rapid bootstrap replicates.

**Table 1:** Sequencing output summary, assembly statistics, and homology search results for *Ixodes scapularis* metagenome libraries. Sequencing output summary, assembly statistics, and homology search results for *Ixodes scapularis* metagenomic raw reads and assembled metagenome scaffolds prepared from Naval Weapons Station Earle (NWSE; top) and Perrineville Lake Park (PVIL; bottom) tick pools. The BLASTn analyses were conducted against the NCBI ’nr’ database + *I. scapularis* ISE6 and Wikel genomes (Miller et al., 2018; Gulia-Nuss et al., 2016). BLASTx analyses were conducted against the NCBI ’nr’ database + predicted proteome of the two tick strains above.

	**# Untrimmed/ trimmed reads**	**BLASTn hits** **(reads)**	**BLASTx hits (reads)**	**# Assembled scaffolds / N50**	**BLASTn hits** **(scaffolds)**	**BLASTx hits** **(scaffolds)**	**# 16S reads**	**# 16S reads overlapped**
**NWSE**	24.54M/24.02M	23.3M (97.1%)	3.70M (15.4%)	751,985/601 bp	746,410 (99.3%)	171,499 (22.8%)	418,528	376,944 (90.0%)
**PVIL**	26.46M/25.91M	25.1M (97.2%)	4.13M (16.0%)	810,827/584 bp	804,722 (99.3%)	179,446 (22.1%)	498,534	461,306 (92.5%)

### Results and Discussion

#### Shotgun sequencing and taxonomic binning

Shotgun sequencing output and homology search results are summarized in Table 1; of the 24.0M and 25.9M respective NWSE and PVIL reads remaining after trimming, 97.1% and 97.2% had BLASTn hits against the ‘nt’ database + *I. scapularis* genome while 15.4% and 16.0% had BLASTx hits against the ‘nr’ protein database. Of the 751,985 and 810,827 respective assembled NWSE and PVIL metagenome scaffolds, 99.3% and 99.3% had BLASTn hits against the ‘nt’ database + *I. scapularis* genome while 22.8% and 22.1% had BLASTx hits against the ‘nr’ protein database. Far fewer matches were found when searching protein databases due to the fact that the majority of our genome data presumably represent non-coding host tick DNA, however this step was used as a secondary source of evidence for taxonomic binning as protein homology may remain conserved for metagenome constituents that do not have a sequenced genome or have diverged at the nucleotide level.

**Figure 1:**
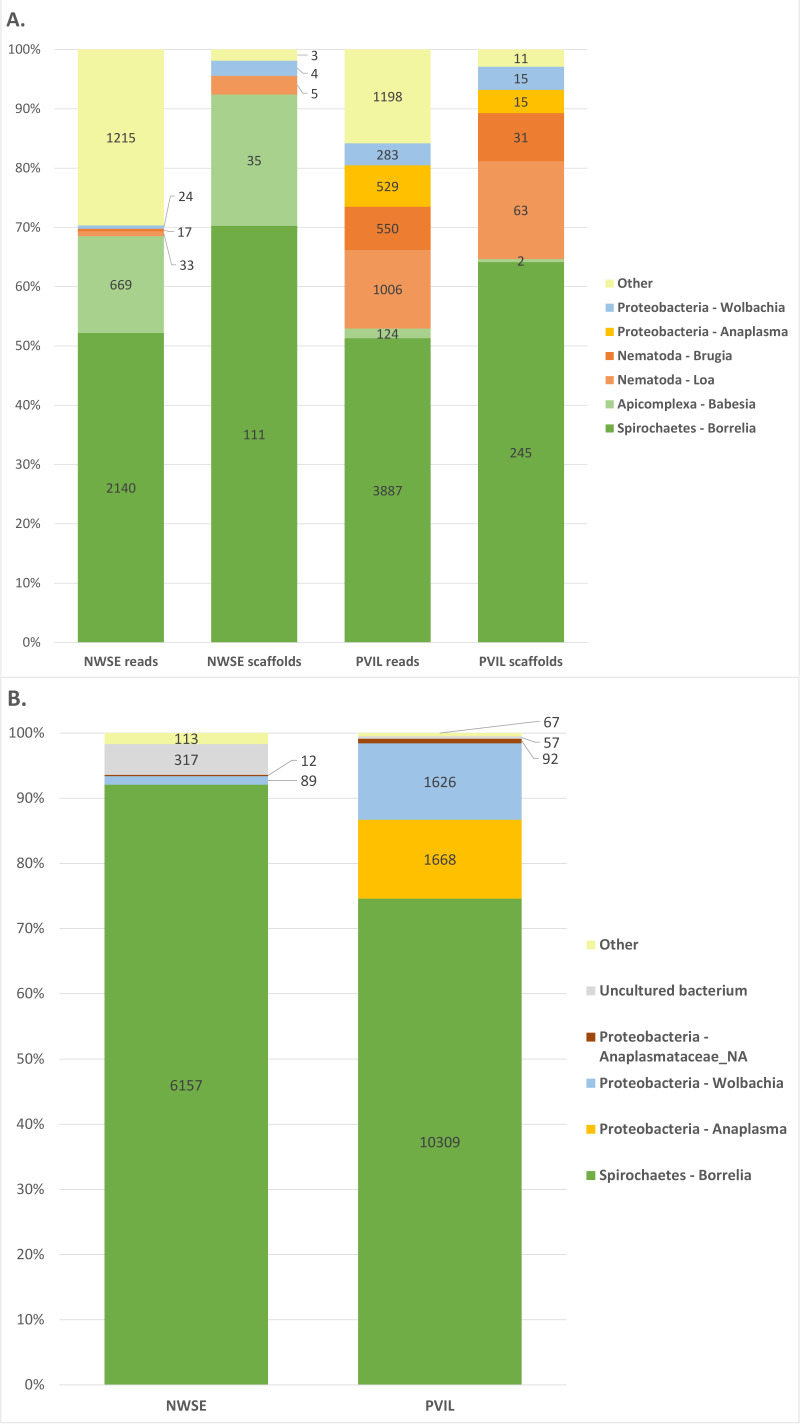
Summary of taxonomic assignments for raw reads, metagenome scaffolds, and 16S amplicon sequencing. Graphical summary of taxonomic assignments for (A) raw read and assembled metagenome scaffolds excluding Arthropoda (host) and *Rickettsia* (symbiont; summarized in Table 2), and (B) 16S amplicon sequencing excluding *Rickettsia* (symbiont; summarized in Table 2).

Examination of the BLASTn output (Fig. 1, Summarized in Table 2 (raw BLAST output and taxonomic annotations in Data S1–S8)) revealed a majority of the raw reads had top hits to ten genera: the proteobacterial genera *Rickettsia*, *Anaplasma* and *Wolbachia*; the spirochaete genus *Borrelia*; the Arthropod (*e.g.*, host tick derived) genera *Ixodes*, *Rhipicentor* and *Ornithodoros*; the apicomplexan genus *Babesia*; and the nematode genera *Loa* and *Brugia*. Inspection of reads derived from tick genera other than *Ixodes* were found to be from conserved genomic loci for which equal-scoring top hits were obtained to multiple species. A similar pattern emerged when analyzing the assembled scaffolds: eight genera (*Rickettsia*, *Anaplasma*, *Wolbachia*, *Borrelia*, *Ixodes*, *Babesia*, *Loa* and *Brugia*) represented all but 14 of the 1.55M total scaffolds with BLASTn hits (Table 2, Data S3 & S4). By contrast, DIAMOND translated protein homology (*i.e.,* BLASTX-like) searches of both reads and scaffolds contained hits to a broader diversity of taxa (Table S1; Data S5 & S8) although further inspection revealed this to be artifactual; a majority of non-coding host tick genomic DNA in the sample, when translated, had weak local alignments to proteins and/or mobile genetic elements of unrelated organisms in the ‘nr’ database. For example, a combined 271,697 reads from both locations had top hits to *Ehrlichia minasensis* (a tick-borne pathogen (Cabezas-Cruz et al., 2016)), however these hits comprised only 39 unique NCBI accessions for which the majority were reverse transcriptase and integrases, and all of which had perfect or near-perfect BLASTn alignments to the *Ixodes scapularis* nucleotide genome sequence thus making them likely retroelements. For this reason, we focused predominantly on the BLASTn results moving forward.

**Table 2:** Summary of BLASTn taxonomic assignments for raw reads and metagenome scaffolds. Homology searches conducted via BLASTn against the NCBI nt nucleotide database using (A; top) the raw reads and (B; bottom) assembled metagenome scaffolds as a query. The mean k-mer coverage for each scaffold as calculated by the SPAdes assembler is shown in parentheses.

**A. Raw reads**						
**NWSE**					**PVIL**	
Arthropoda - *Ixodes*	23243128				Arthropoda - *Ixodes*	25043992
Proteobacteria - *Rickettsia*	40522				Proteobacteria - *Rickettsia*	58848
Spirochaetes - *Borrelia*	2140				Spirochaetes - *Borrelia*	3887
Arthropoda - *Rhipicentor*	761				Nematoda - *Loa*	1006
Arthropoda - *Ornithodoros*	743				Arthropoda - *Rhipicentor*	611
Apicomplexa - *Babesia*	669				Nematoda - *Brugia*	550
Nematoda - *Loa*	33				Proteobacteria - *Anaplasma*	529
Proteobacteria - *Wolbachia*	24				Arthropoda - *Ornithodoros*	516
Nematoda - *Brugia*	17				Proteobacteria - *Wolbachia*	283
Other	1215				Arthropoda - *Haemaphysalis*	180
					Apicomplexa - *Babesia*	124
					Other	1198
Total hits	23289252				Total hits	25111724
				
**B. Metagenome scaffolds**						
**NWSE # scaffolds (mean** ***k-mer*** **coverage)**					**PVIL # scaffolds (mean** ***k-mer*** **coverage)**	
Arthropoda - *Ixodes*	746118 (3.57x)				Arthropoda - *Ixodes*	804221 (8.9x)
Proteobacteria - *Rickettsia*	134 (12.81x)				Spirochaetes - *Borrelia*	245 (1.49x)
Spirochaetes - *Borrelia*	111 (1.27x)				Proteobacteria - *Rickettsia*	119 (19.73x)
Apicomplexa - *Babesia*	35 (1.05x)				Nematoda - *Loa*	63 (0.99x)
Nematoda - *Loa*	5 (0.86x)				Nematoda - *Brugia*	31 (1.05x)
Proteobacteria - *Wolbachia*	4 (0.92x)				Proteobacteria - *Anaplasma*	15 (1.01x)
Other	3				Proteobacteria - *Wolbachia*	15 (1.21x)
					Apicomplexa - *Babesia*	2 (0.92x)
					Other	11
Total hits	746410				Total hits	804722

#### 16S taxonomic diversity

Our 16S amplicon sequencing produced 418,528 paired reads from the NWSE library and 498,534 paired reads from the PVIL library. After quality trimming and primer removal, 376,944 (90.0%) and 461,306 (92.5%) respective NWSE and PVIL read pairs were overlapped into a single fragment prior to analysis (Table 1). All merged amplicons had BLASTn hits to the NCBI ‘nr’ database (Summarized in Fig. 1 and Table S2; raw BLAST output in Table S3). The taxonomic composition of these hits mirrored those of the shotgun metagenome sequencing, as 99.8% of the NWSE 16S fragments were assigned to the genera *Rickettsia*, *Borrelia*, and *Wolbachia*, while 99.90% of the PVIL fragments were assigned to the same three genera with the addition of *Anaplasma*.

Both shotgun and 16S amplicon sequencing identified the same set of bacterial taxa (*Rickettsia, Borrelia, Wolbachia,* and *Anaplasma*) as the major constituents, indicating (1) that the shotgun sequencing approach identified bacterial constituents despite the much greater abundance of tick DNA, and (2) that the two *I. scapularis* pools examined here contain few other microbial genera. Recent experiments employing surface sterilization, tissue dissection and/or rigorous statistical analyses have found that *I. scapularis* and *I. pacificus* (as well as species of *Amblyomma* and *Dermacentor*) tend to harbor only a limited number of permanent bacterial species, primarily endosymbionts (Lado et al., 2020; Pavanelo et al., 2020; Couper et al., 2019; Ross et al., 2018). This is reflected in the fact that genomes of many tick-associated endosymbionts and pathogens have lost suites of effector-immunity genes that mediate inter-bacterial competition, suggesting that these microbes encounter a limited community diversity within the tick (Ross et al., 2018).

Given the agreement between taxonomic affinity of shotgun genome data generated and 16S taxonomic assignment using BLASTn (the *Wolbachia* 16S BLASTn hits for example were >99.5% identical spanning a local alignment of >400 nt (Table S3)), we chose to present representative data from only the BLAST analyses herein.

### *I. scapularis* mitochondrial DNA variants

**Figure 2:**
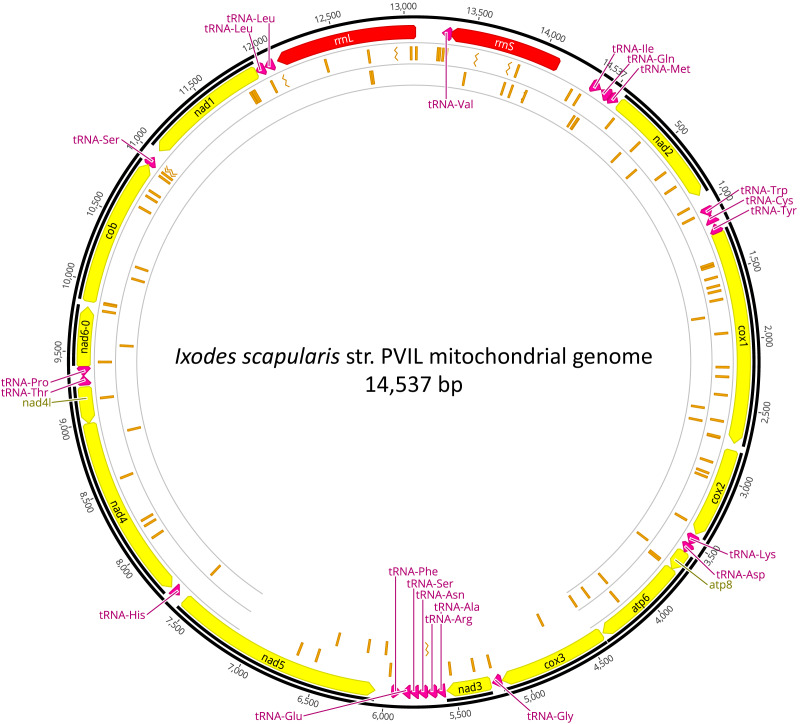
Mitochondrial genome of *Ixodes scapularis*. The annotated mitochondrial genome (14,537 bp) of *Ixodes scapularis* assembled from Illumina short-read sequence data derived from the Perrineville (PVIL) sample. The outer ring (ring #1) illustrates coding (CDS) sequence annotated in yellow, ribosomal RNA loci in red and tRNA genes in magenta; ring #2 illustrates locations of single nucleotide variants present in shot-read mapping data (>25% by depth) that occur in the NWSE data to the exclusion of the PVIL data; inner (ring #3) illustrates locations of single nucleotide variants present in shot-read mapping data (>25% by depth) that occur in the PVIL data to the exclusion of the NWSE data. Chevrons indicate positions of indels.

An overwhelming number of the short-reads were derived from the *I. scapularis* genome, as 23.24 M (99.80%) NWSE reads and 25.04M (99.73%) PVIL reads had top hits to various *Ixodes* genomic DNA scaffolds or Genbank nucleotide sequences (Table 2). As described in the Methods section, we were able to assemble a complete *I. scapularis* mitochondrial genome reference sequence. This 14,537 bp circularized genome sequence (Fig. 2) representative of the PVIL sample data was sequenced at a mean depth of 352x coverage and spanned 100% of the *Ix. ricinus* mitochondrial genome sequence (GenBank accession NC_018369.2) and has been deposited to GenBank under accession number MZ645749). Like other tick mitochondria, the organelle encodes 13 protein-coding genes, 22 tRNAs, and two ribosomal RNA genes (Table S4). A BLASTn homology search of the NCBI ‘nt’ nucleotide database indicates that the top-scoring complete mitochondrial genome is that of *Ixodes ricinus* strains IR_63 (KF197121) and IR_25 (KF197116), each with 87.03% sequence identity across 100% of the complete genome. The gene order was conserved with all 14 completely sequenced mitochondria currently available from genus *Ixodes* and follows the template of Wang et al. (2019). We searched this reference for segregating nucleotide variants that may be indicative of reproductive isolation and/or structure between the two tick populations sampled here (Table S5). Of the 112 total SNVs occurring at >25% frequency in the NWSE sample (Fig. 2), 67 (59.8%) were unique and were not present in the PVIL data; five resulted in amino acid substitutions. Of 75 SNVs present in the PVIL data (*i.e.,* minority polymorphisms present in the raw read data, as the consensus that we mapped to was generated from PVIL data (above)), 29 (38.7%) were unique and not present in the NWSE data; three resulted in amino acid substitutions. Of note, a particular set of variants was identified within the PVIL 12S rRNA locus at position 13,984 (Fig. S1) in which 66.5% of the reads (*n* = 268, total coverage = 403x) carried either a +A (*n* = 132) or +ATA (*n* = 134) insertion that was absent from the NWSE data (NWSE coverage = 315x). As the rRNA is a non-coding structural molecule, these SNVs do not confer an amino acid substitution. The extent to which these variants occur in Perrineville and New Jersey ticks in general will require more sampling, however the data indicate that multiple mitochondrial haplotypes may exist across a relatively small scale (PVIL and NWSE collection sites are separated by approximately 23 km) that may be of future utility in genotyping and population structure analyses. We focused only on mitochondrial variants as the MiSeq output (when coupled with v3 sequencing reagents) is ca. 15 Gbp per flowcell and provides insufficient depth of the ca. 2.22 Gbp *I. scapularis* haploid genome (Miller et al., 2018) to confidently infer SNVs at single-copy chromosomal loci in our samples.

#### Rickettsia endosymbiont

In each sample, the majority of non-tick reads (90.1% and 88.6% from NWSE and PVIL, respectively) had top hits to the proteobacterial genus *Rickettsia* (Table 2) and most likely derive from the known *I. scapularis* endosymbiont *Rickettsia buchneri* (Kurtti et al., 2015). The greatest number of *Rickettsia* reads hit the draft genome sequence of *Rickettsia monacensis* (NCBI accession LN794217), a spotted fever pathogen, while the majority of DIAMOND hits (167,949/365,340) were to *Rickettsia endosymbiont of Ixodes scapularis* (REIS; a synonym of *Rickettsia buchneri* (Kurtti et al., 2015; discussed below) Data S5 & S6), followed by *R. amblyommatis* and *R. buchneri.* We attribute this to exclusion of the *R. buchneri* symbiont genome from the ‘nt’ nucleotide database coupled with the presence of a predicted proteome in ‘nr’. To test this, both NWSE and PVIL libraries were mapped to the genomes of *R. monacensis* and *R. buchneri* str. ISO7 (NCBI accession GCF_000696365.2) concurrently, with non-specific hits (those mapping equally well to both genomes) discarded. Of the 200,410 and 298,528 reads aligned for each respective library, only 3,089 (1.5%) and 4,183 (1.4%) were to the *R. monacensis* genome. The reads that mapped to the *R. buchneri* genome had an average pairwise identity of 99.2%, while those mapping to *R. monacensis* had a pairwise identity of only 89.4% and are unlikely to be derived from this species. Taken together, these results allow us to conclude that the *Rickettsia* strain recovered in our *Ixodes* populations is *R. buchneri*.

*Rickettsia* endosymbionts are common among *Ixodes* spp. and there is evidence that they share a coevolutionary past (Díaz-Sánchez et al., 2019). *Rickettsia buchneri* has been documented at a high prevalence in many *I. scapularis* populations from the Northeastern US (*e.g.*, 46.0% in Maine, 64.9% in Pennsylvania, 65%–100% in New York, and 93.3% in Connecticut) (Moreno et al., 2006; Pokutnaya et al., 2020; Steiner et al., 2014; Tokarz et al., 2019). Its occurrence is much higher in female *I. scapularis* than in males, localizing primarily within the ovaries where it is passed vertically to larvae (Kurtti et al., 2015; Zolnik et al., 2016). While endosymbionts are known to have a range of impacts on their tick hosts (Bonnet et al., 2017) information on the impact of *R. buchneri* infection on *I. scapularis* is sparse. However, some studies have indicated that it may provide them with folate (Hunter et al., 2015) and alter their motility (Kagemann & Clay, 2013). It is also possible *R. buchneri* could affect the susceptibility of *I. scapularis* to colonization by pathogens, as has been observed for *R. bellii* in *Dermacentor andersoni* (Gall et al., 2016).

To qualify our *R. buchneri* reads further, we mapped short-reads from both locations (NWSE and PVIL) to the *Rickettsia endosymbiont of Ixodes scapularis* str. Wikel genome sequence + 3 associated plasmids (NCBI BioProject PRJNA33979). We used this strain as opposed to str. ISO7 (above) as it was assembled to a single chromosome. Results indicate that 199,787 reads (ca. 21x avg. coverage of the reference) from the NWSE library and 299,315 reads (ca. 32x avg. coverage) from the PVIL library align to the symbiont genome. Using this short-read mapping, we detected and manually curated 19 single-nucleotide variants (SNVs; Table S6) present in the symbiont genome data; six variants occurred at >90% frequency in the NWSE sample (three were fixed at 100%) while present in <25% of the reads mapping to the equivalent locus in the PVIL library (three were absent entirely at 0%). This analysis also uncovered two diverged genomic regions in the symbiont genome of each sample, both of which occur in genes encoding conjugative transfer proteins. One region spans nucleotides 706,940–706,952 (RAGE-Be) of the symbiont chromosome scaffold CM000770 in which the PVIL read data support seven linked polymorphisms at high coverage that are absent from the NWSE data (Fig. S2). All seven variants are synonymous and occur within the conjugal transfer pilus assembly protein *TraU* gene. The second region spans nucleotides 760,409–760,434 (RAGE-F) of the genome scaffold and contains six linked SNVs that are fixed in the NWSE data but occur only at low coverage in the PVIL library (Fig. S3). Four polymorphisms are non-synonymous and result in four amino acid changes in the conjugative transfer protein *TraA* gene.

Both *TraU* and *TraA* are members of a 21-gene integrative conjugative element known as the Rickettsiales amplified genetic element (RAGE; Gillespie et al., 2012). RAGE elements together encode an F-like type IV secretion system allowing translocation of genetic material between diverse intracellular bacteria, and have been discovered on plasmid or chromosomal DNA of tick-borne *Rickettsia bellii*, *R. massiliae* and *R. tamurae* species, as well as *R. peacockii* and *R. felis* (Hagen et al., 2018). *Rickettsia buchneri* however is the only known species found to encode multiple RAGE elements (7 chromosomal and two encoded on plasmids) where they account for 9% of the genome by length (Gillespie et al., 2012; Hagen et al., 2018). The variants described above occur in two such elements, the *TraU* gene of RAGE-Be and *TraA* gene of RAGE-F, using the naming scheme of Gillespie et al. (2012). Both variation within and loss of the genes that constitute the RAGE-T4SS module have been reported between the two *R. buchneri* strains with currently sequenced genomes, as well as within natural tick populations (Hagen et al., 2018). To test whether the variants described here were mapping artifacts caused by the lack of a particular *TraA/U* element within the reference resulting in secondary read alignment to a sub-optimal location, we repeated the analysis using the *R. buchneri* strain ISO7 genome reference (Kurtti et al., 2015) with identical results (not shown). These data indicate concerted evolution acting on individual components of the RAGE-T4SS in *I. scapularis* symbiont genomes at the host (tick) population level.

#### Pathogens and other bacteria

In addition to the rickettsial endosymbiont, our metagenome sequencing also identified 2,140 NWSE and 3,887 PVIL reads with top hits to the spirochaete genus *Borrelia* (Table 2), with a majority (38.2% and 44.3% respectively) hitting *B. burgdorferi* strain MM1, originally isolated in Minnesota (Jabbari et al., 2018; Loken et al., 1985) and associated plasmids (Data S1 & S2). Short-read mapping aligned 1,841 and 3,248 reads to the *B. burgdorferi* strain MM1 genome. This causative agent of Lyme disease is known to be locally common in these tick populations from prior studies at the same sites, with prevalence ranging from 43.5–52.7% of adults infected (Dolan et al., 2016; Schulze et al., 2013). We did not detect *B. miyamotoi* in either pool*,* which is known to be rare across NJ (∼2.7% infected) (Egizi et al., 2018), nor *B. mayonii*, which has so far only been found in the Midwest (Kingry et al., 2017).

A second potential human pathogen was recovered from the PVIL library only, as 529 reads were found to have top hits to the proteobacterial genus *Anaplasma* (Table 2). Of these, 401 hits were to *A. phagocytophilum* strain Dog2 (an agent of Anaplasmosis originating from a MN dog (Al-Khedery et al., 2012); Genbank accession GCA_000439795, Data S1 & S2). No DNA corresponding to this pathogen was identified within the NWSE library. While *A. phagocytophilum* is also known to occur locally, its published infection prevalence in adult *I. scapularis* is substantially lower than that of *B. burgdorferi*, which could explain why it was not detected in both samples. Past work has shown that around 20–22% of adult *I. scapularis* are infected with *A. phagocytophilum* in Monmouth County (Dolan et al., 2016; Egizi et al., 2018). Two primary strains of *A. phagocytophilum* have been identified in *I. scapularis*, one pathogenic to humans (Ap-ha) and one that has not been associated with human illness and primarily circulates in white-tailed deer (Ap-V1), differing by two SNPs in the 16S rRNA gene (Krakowetz et al., 2014). While the single 16S read available in our dataset contained the Ap-ha associated SNPs, we caution that a single read could contain errors and is likely not definitive for strain assignment. However, we were able to verify that the Dog2 strain genome, the closest match in Genbank to our reads, is the Ap-ha strain.

**Figure 3:**
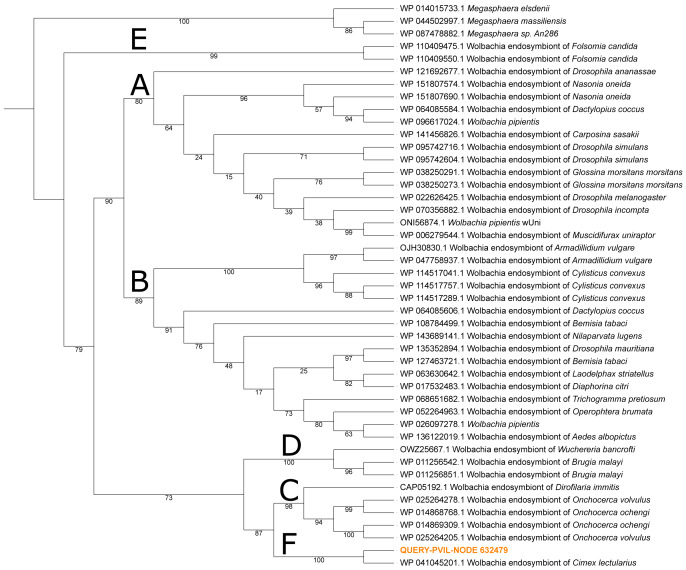
Maximum-likelihood phylogeny of *Wolbachia* spp. constructed from elongation factor Tu (*TufA*) protein sequence alignment. The homolog recovered in our *Ixodes scapularis* metagenome sequencing (QUERY-PVIL-NODE_632479) in orange text. Nodal support values are the result of 2,000 rapid bootstraps; the major clades of Brown et al. (2016) are labeled A–F. The tree is represented as a cladogram without branch lengths.

The final bacterium with tangible representation in our data belonged to the proteobacterial genus *Wolbachia*, the top hit to 24 NWSE and 287 PVIL metagenome reads (Table 2), with 91.7% and 89.2% of respective hits to the *Wolbachia* symbiont of *Cimex lectularius* (the common bed bug) strain wCle genome sequence (Data S1 & S2). *Wolbachia* is commonly an endosymbiont of many hematophagous arthropods (*e.g.*, mosquitoes) estimated to infect up to 60% of known insect species (De Oliveira et al., 2015). To determine the taxonomic placement of the *Wolbachia* found in this study, we constructed a maximum-likelihood phylogenetic tree using all available *Wolbachia* hits within the ‘nr’ database to the translated 152 amino acid ORF present on PVIL assembled scaffold NODE_632479, which encodes an elongation factor Tu (EF-Tu) protein that maintains 97.4% amino acid sequence identity over the entire 457 bp scaffold. This tree (Fig. 3) mirrors the branching order of Brown et al. (2016) and places the query sequence sister to *Wolbachia* strain wCle in a clade of obligate mutualists (clade F of Brown et al. (2016)). Zhang, Norris & Rasgon (2011) reported a novel *Wolbachia* species from *Amblyomma americanum* ticks collected in Maryland, and constructed a five-gene phylogeny that also placed this bacterium in an F-group clade containing the *Cimex lectularius* symbiont. We were unable to place our *Wolbachia* strain in this tree due to limited genome data recovery of appropriate homologs. At least one prior study posited that the *Wolbachia pipientis* strain detected in their *Ixodes ricinus* tick sample was due to cryptic presence of an *Ixodiphagus hookeri* endoparasitoid wasp (Plantard et al., 2012), however we did not detect hymenopteran DNA in our samples, nor was our *Wolbachia* strain closely related to *W. pipientis*.

If residing in the tick itself, the occurrence of *Wolbachia* is of particular interest due to known impacts of congeners on the reproductive fitness and vector competence of mosquitoes (Caragata, Dutra & Moreira, 2016). These discoveries have led to strategies of controlling mosquito-borne diseases by manipulating *Wolbachia* infectivity (paratransgenesis). Further characterization of tick-endosymbiont interactions could facilitate the use of paratransgenic strategies to control tick-borne diseases (Díaz-Sánchez et al., 2019).

#### Eukaryotic sequences

Multiple eukaryote constituents were identified within our metagenome data. Host tick (*I. scapularis*) DNA expectedly constituted an overwhelming majority of the assembly given the derivation of the tissue sample and large (2.22 Gbp) genome size of the tick with respect to other microbial and symbiont genomes present in the sample; when coupled with a relatively low-output sequencing run as described above, this would result in a fragmented assembly and large number of contigs as witnessed here.

Reads derived from the Apicomplexan parasite *Babesia* were identified in both samples (Table 2), with 669 hits in the NWSE library and 124 from the PVIL data. *Babesia microti* strain RI was the predominant taxon represented in both libraries (71.2% and 98.4% of each respective subset of reads). Like *B. burgdorferi* and *A. phagocytophilum*, *Babesia microti* is also frequently detected in Monmouth County ticks. In a prior field study, 8.2% of *I. scapularis* adults from PVIL and NWSE were positive for *Ba. microti* (Schulze et al., 2013). While a single NWSE amplicon (BMY3G:1:2110:24100:13578 (Supplemental File 1)) was identified with 100% homology exclusively to the *Ba. microti* apicoplast 16S rRNA locus and a further 489 reads had hits to *Ba. microti* with an average nucleotide similarity score of 99.5%, there was also a moderate proportion of hits to *Babesia divergens* (*n* = 148; Data S1) with a much lower average similarity score (86.9% nucleotide homology). A manual BLASTn into the publicly available and fully sequenced *Ba. microti* genome using these reads as a query returned no hits, and thus they appear to indicate a second species of *Babesia* is present in our dataset. Proper identification of these reads will likely require follow-up PCR with gene-specific primers, as there were no perfect or near-perfect matches in the NCBI ‘nt’ database and most *Babesia* species in public gene databases are represented by few (often non-overlapping) loci that prevent a broad phylogenetic analysis with homologs identified in our sequencing. One parsimonious possibility may be *Babesia odocoilei*, as this protozoal parasite of cervids is known to occur in I*. scapularis* ticks throughout the Northeastern US (Steiner et al., 2014) and for which a paucity of sequence exists in public databases.

Nematodes represent another eukaryotic component within our sample. We identified 59 and 1,694 reads with top hits to nematodes in the NWSE and PVIL libraries, of which 88.1% and 94.3% were within the family Onchoceridae (Table 2). Three amplicons (six paired reads) were identified with hits to nematode ribosomal RNA, of which one pair maintained 100% nucleotide homology over >300 nt to the small subunit of *Filarioidea sp. ex Ixodes scapularis* RTS-265 (NCBI accession MK868471.1) reported recently from *I. scapularis* ticks in New York and Connecticut (Tokarz et al., 2019). The remaining two amplicons were derived from the large subunit rRNA for which no data exist for the latter species, and thus the hits share lesser homology to various species.

Nematodes have previously been detected in *Ixodes scapularis* from Wisconsin (Cross et al., 2018) and Connecticut (Namrata et al., 2014) and appear to be closely related to the above accession from Tokarz et al. (2019) as well as to nematodes from *A. americanum* collected in Maryland and Virginia (Henning et al., 2016; Zhang, Norris & Rasgon, 2011). This clade of tick-associated nematodes is usually sister to *Acanthocheilonema* spp. (Namrata et al., 2014; Tokarz et al., 2019) or *Monanema*, when included, as in Henning et al. (2016); here their closest match is to *Monanema* spp. These detections are based on DNA sequencing and not physiological observation, thus their role in the tick (as potential commensals or tick-borne pathogens) remains unknown. In Europe, the brown dog tick *Rhipicephalus sanguineus* is an intermediate host of canine infectious filaria (Brianti et al., 2012; Olmeda-Garcia, Rodriguez-Rodriguez & Rojo-Vazquez, 1993; Otranto et al., 2012).

Both Tokarz et al. (2019) and Cross et al. (2018) attribute the *Wolbachia* they detected in their samples to filarial nematodes; while nematodes in the genus *Acanthocheilonema* have not been found to contain *Wolbachia*, there is evidence of a historical association: lateral gene transfer from *Wolbachia* into the *Acanthocheilonema* genome (Keroack et al., 2016; Mcnulty et al., 2010). Conversely, nematodes in the genus *Monanema* have not been found to carry *Wolbachia* (Ferri et al., 2011). Further work is needed to characterize this potentially novel clade of nematodes in US-based *Ixodes* and *Amblyomma* ticks and to determine whether the detected *Wolbachia* is carried by ticks or by their associated nematodes.

## Conclusions

Intriguingly, three of the most commonly reported *I. scapularis* associated human pathogens (*B. burgdorferi, A. phagocytophilum, Babesia microti)* were detected in just two pools of six ticks each. The diseases transmitted by these pathogens are the three most common vector-borne diseases in Monmouth County: in 2018, there were 506 Lyme disease cases, 27 Babesiosis cases and 11 Anaplasmosis cases (Njdoh, 2018). In addition to these pathogens, we also obtained data on a variety of other eukaryotic and prokaryotic holobiont constituents, including a rickettsial endosymbiont, *Babesia* species, and *Wolbachia* (F-group). Additional work is needed to evaluate the interactions among tick-borne microbes including potential strategies for tick population control by manipulating their endosymbionts (Bonnet et al., 2017).

Both shotgun metagenomic sequence analysis and 16S amplicon sequencing highlighted the same microbial genera, and our finding of a very sparse set of microbes within the tick pools examined (consisting primarily of endosymbionts and pathogens) stands in stark contrast to other arthropod microbiomes *e.g.*, in mosquitoes (Gao et al., 2019; Scolari, Casiraghi & Bonizzoni, 2019; Strand, 2018) but in agreement with a growing number of recent tick studies (Ross et al., 2018; Couper et al., 2019; Tokarz et al., 2019; Lado et al., 2020). While there is evidence that environmental microorganisms may be transiently acquired, the tick microbiome appears to converge over time on the same minimal suite of core species (Couper et al., 2019; Hawlena et al., 2013). As a result, the number of potential microbes that could be interacting with pathogens within the tick’s gut and potentially affecting their transmission is also limited. This directly impacts research focusing on manipulation of the tick microbiota to study the effects of individual species on infection and transmission, as well as co-evolution of ticks and their symbionts (reviewed in Díaz-Sánchez et al., 2019).

Given our small sample size, this study was not intended to be a pathogen survey nor to estimate the prevalence of these agents in the tick populations sampled. Rather, our primary goal was to evaluate the utility of shotgun sequencing as a tool to compare hologenome data from two samples of ticks—and to determine whether there are any observable differences at a relatively small spatial scale, whether in the host genome, the symbiont, or associated bacteria. While the pooled approach chosen to minimize costs in this exploratory analysis prevented calculation of haplotype frequencies and frequency-dependent genetic distance metrics, we were able to qualitatively examine differences between the populations. In particular, a large majority of mtDNA variants were not shared between samples; this is intriguing given the relatively small distance between collection sites. Observed population structure of *Ixodes scapularis* has been reported as high (Araya-Anchetta et al., 2015) with barriers to gene flow observed at regional levels using mitochondrial polymorphisms (Trout, Steelman & Szalanski, 2009). However, examinations of genetic differentiation at the intra-county level are sparse compared to broader-scale investigations at the state level or above. Our results provide compelling justification for pursuing further investigations at a localized spatial scale, as well as the possibility of using endosymbiont genetic markers as additional lines of evidence in tick phylogeographic studies.

Our analysis cannot be considered a population-level approach as would be conducted using a larger number of individuals and *e.g.*, pool-seq (reviewed in Schlötterer et al., 2014) and results can be biased by factors such as incomplete sampling of the population, heteroplasmy (or leakage of paternal mitochondria to the zygote, a phenomenon observed in multiple tick genera (Mastrantonio et al., 2019; Xiong et al., 2013), and nuclear-mitochondrial transpositions (NUMTs (Gaziev & Shaikhaev, 2010)). The NUMT scenario is less likely given that the high coverage of each SNV in our data (Table S5) would not be expected for low-copy nuclear-encoded loci in a 2.22 Gbp tick genome given our sequencing output. Commensurately, deeper sequencing and a more expansive sampling of individuals from both populations would be required to accurately infer variation of alleles that are not fixed, *i.e.,* in conducting a pool-seq population-level analysis as described above.

Fine-scale genetic discrimination among tick populations is an attractive target for illuminating the complex spatial epidemiology of tick-borne diseases. Genetically disparate populations of ticks could differ in acaricide resistance, host preference, phenology, vector competence or other epidemiologically relevant factors (Esteve-Gassent et al., 2016) and thus delineating the spatial scale at which tick populations remain connected has implications for the spread and control of tick-borne pathogens.

##  Supplemental Information

10.7717/peerj.12313/supp-1 Supplemental Information 1 Illustration of nucleotide variants within the mitochondrial 12S rRNA locus Illustration of short-read mapping of sequence data to the *Ixodes scapularis* consensus mitochondrial genome generated in this study. Polymorphisms are hilighted. Data from (A; top) the NWSE tick sample lack the variants prevalent within the (B; bottom) PVIL data.

10.7717/peerj.12313/supp-2 Supplemental Information 2 Illustration of nucleotide variants within the *Rickettsia buchneri* RAGE-Be element Illustration of short-read mapping of sequence data to the *Rickettsia endosymbiont of *Ixodes scapularis** str. Wikel genome (NCBI BioProject PRJNA33979. The region spanning nucleotides 706,940 –706,952 (RAGE-Be element) of the symbiont chromosome scaffold CM000770 is identical to the reference genome with regard to (A; top) the NWSE data however contain seven linked polymorphisms in (B; bottom) the PVIL data. All seven variants are synonymous and occur within the conjugal transfer pilus assembly protein *TraU* gene.

10.7717/peerj.12313/supp-3 Supplemental Information 3 Illustration of nucleotide variants within the *Rickettsia buchneri* RAGE-F element Illustration of short-read mapping of sequence data to the *Rickettsia endosymbiont of *Ixodes scapularis** str. Wikel genome (NCBI BioProject PRJNA33979. The polymorphic region spans nucleotides 760,409 –760,434 of the genome scaffold and contains six linked SNVs (hilighted) that are fixed in the (A; top) NWSE data but occur only at low coverage in the (B; bottom) PVIL library. Four polymorphisms are non-synonymous and result in four amino acid changes (illustrated atop each respective alignment) in the conjugative transfer protein *TraA* gene.

10.7717/peerj.12313/supp-4 Supplemental Information 4 Summary of DIAMOND protein homology taxonomic assignments Homology searches were performed against the NCBI ’nr’ protein databases using raw reads (left) and assembled scaffolds (right). The corresponding top-hit accession numbers were annotated with assigned NCBI taxonomy using taxonomizr and summed for each genus in the “count” column.

10.7717/peerj.12313/supp-5 Supplemental Information 5 Summary of 16S merged amplicon taxonomic assignments Homology searches performed using BLASTn against the NCBI ’nt’ nucleotide database with NWSE (left) and PVIL (right) merged amplicon reads (raw BLAST output in Table S3). The corresponding top-hit accession numbers were annotated with assigned NCBI taxonomy using taxonomizr and summarized here.

10.7717/peerj.12313/supp-6 Supplemental Information 6 Tabular BLASTn output for 16S merged amplicon reads Homology searches performed using BLASTn against the NCBI ’nt’ nucleotide database with NWSE (left) and PVIL (right) merged 16S amplicon reads. The corresponding top-hit accession numbers were annotated with assigned NCBI taxonomy using taxonomizr and appended to BLAST output. These data are summarized in Table S2.

10.7717/peerj.12313/supp-7 Supplemental Information 7 *Ixodes scapularis* mitochondrial genome annotation The mitochondrial genome generated in this study was derived from the Perrineville (PVIL) data, annotated with MITOS (Bernt et al., 2013) and deposited as NCBI accession MZ645749.

10.7717/peerj.12313/supp-8 Supplemental Information 8 Single nucleotide variants identified in *Ixodes scapularis* mitochondrial genome data Variants with a frequency of greater or equal to 25% shown; frequency defined as the number of reads containing the variant divided by total number of reads mapped at each reference nucleotide position. Variant *p*-value of each SNV was defined as the probability of encountering the variant by chance due to sequencing error after averaging base quality scores for all nucleotides that comprise the variant and reference sets separately (as implemented in Geneious Prime 2020 [https://www.geneious.com ]).

10.7717/peerj.12313/supp-9 Supplemental Information 9 Single nucleotide variants identified in *Rickettsia buchneri* genome data Reads were mapped to the *Rickettsia endosymbiont of Ixodes scapularis* str. Wikel genome sequence + 3 associated plasmids (NCBI BioProject PRJNA33979) using BBmap. Variants with a frequency of greater or equal to 25% shown; frequency defined as the number of reads containing the variant divided by total number of reads mapped at each reference nucleotide position. Variant *p*-value of each SNV was defined as the probability of encountering the variant by chance due to sequencing error after averaging base quality scores for all nucleotides that comprise the variant and reference sets separately (as implemented in Geneious Prime 2020 [https://www.geneious.com ]).

## Additional Information and Declarations

### DNA Deposition

The following information was supplied regarding the deposition of DNA sequences: The short-read data generated here are available at the NCBI Sequence Read Archive (SRA): PRJNA690601.

### Data Availability

The following information was supplied regarding data availability: The raw BLAST and/or DIAMOND output, phylogenetic trees and alignments, and assembled metagenome scaffolds are available at:
http://acari.rutgers.edu/data/scapularis/.
