# Supplementary material for: Comparative hologenomics of two Ixodes scapularis tick populations in New Jersey
Source: PeerJ. 2021 Nov 9;9:e12313. doi: 10.7717/peerj.12313 (PMC8588856; doi:10.7717/peerj.12313)
Supplement: Supplemental Information 1 — Illustration of short-read mapping of sequence data to the Ixodes scapularis consensus mitochondrial genome generated in this study. Polymorphisms are hilighted. Data from (A; top) the NWSE tick sample lack the variants prevalent within the (B; bottom) PVIL data. [file peerj-09-12313-s001.pdf]

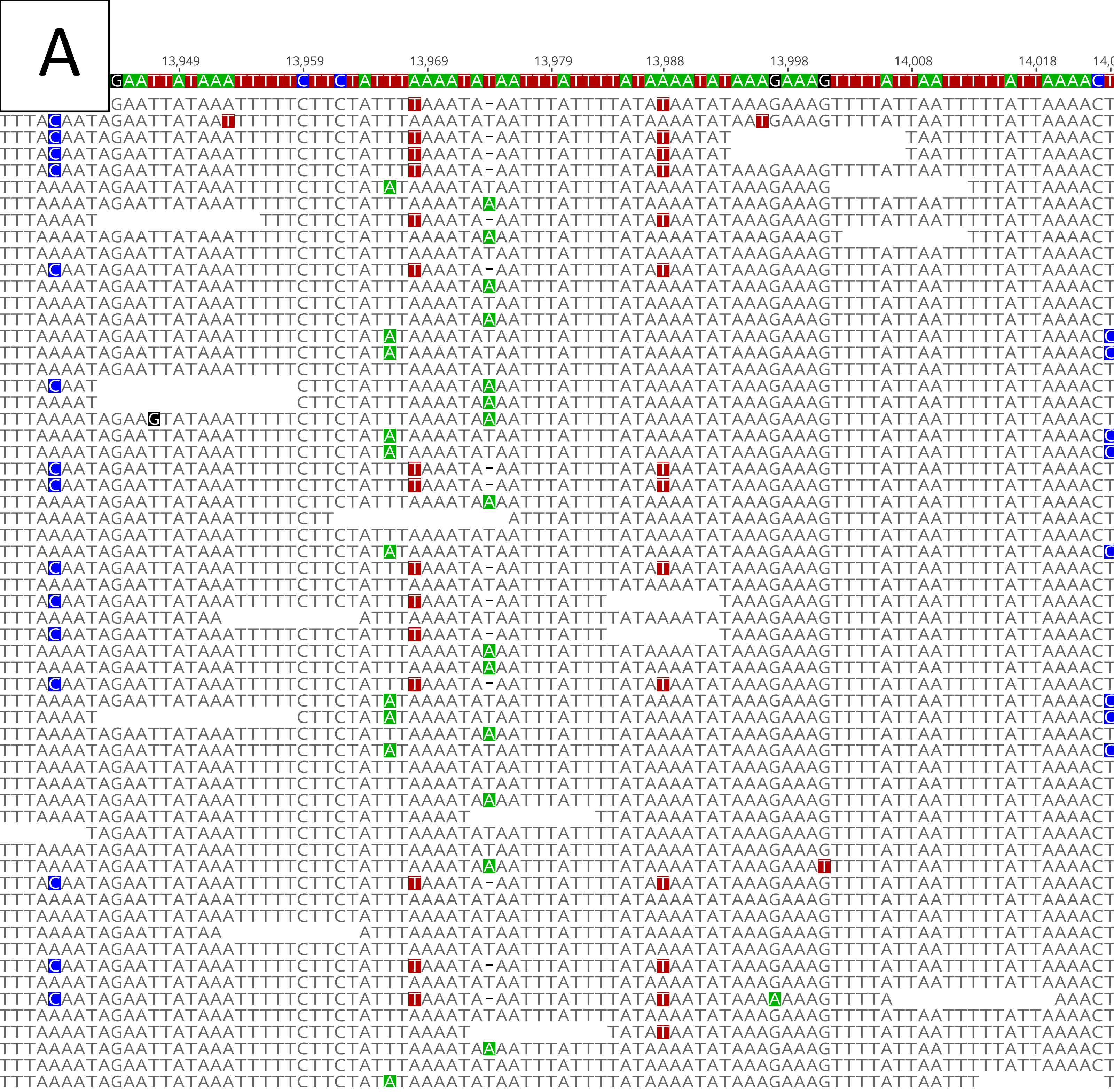
A
13,949
13,959
13,969
13,979
13,988
13,998
14,008
14,018

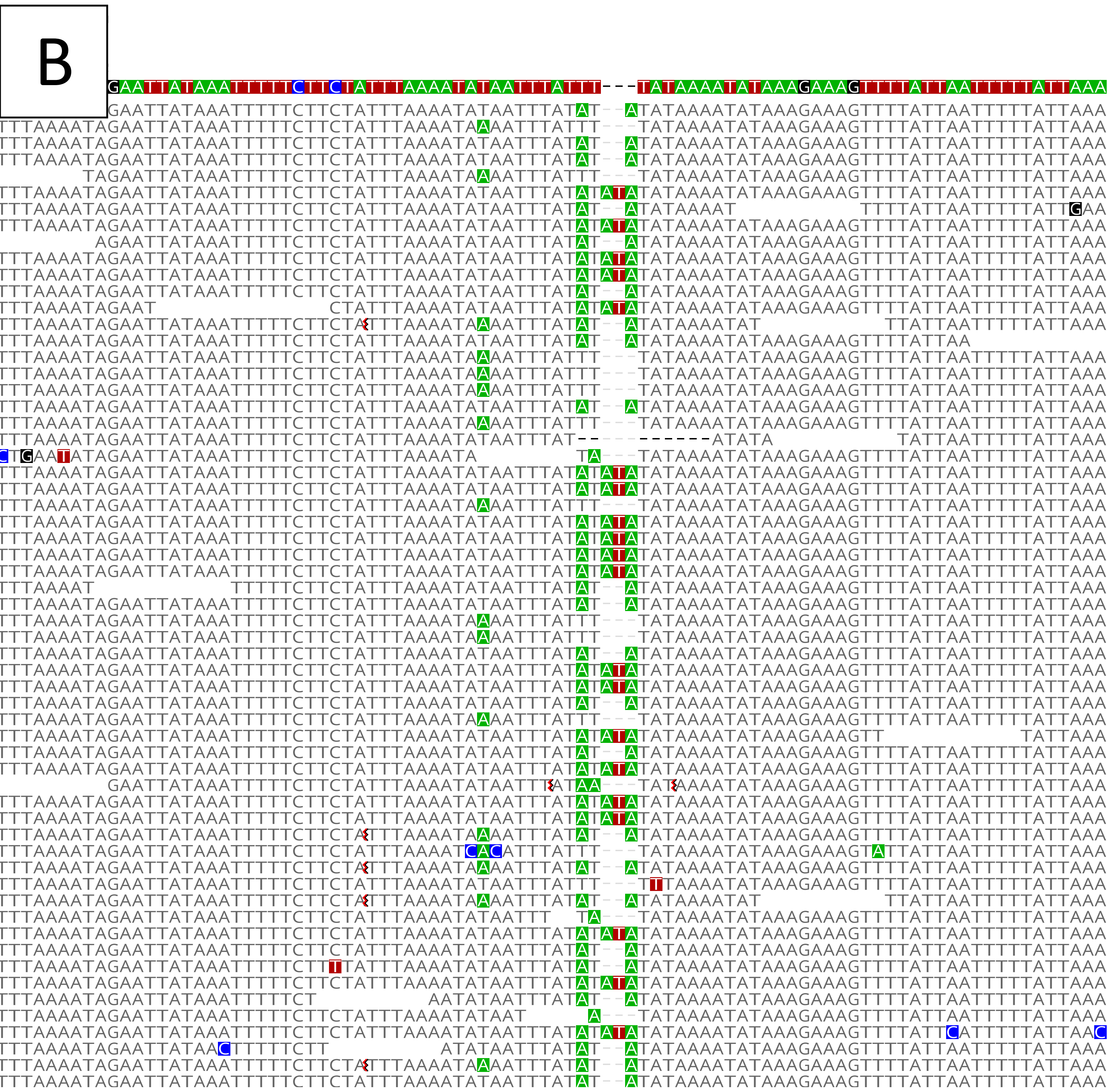
B
