# Supplementary figures and images for: Comparative hologenomics of two Ixodes scapularis tick populations in New Jersey
Source: PeerJ. 2021 Nov 9;9:e12313. doi: 10.7717/peerj.12313 (PMC8588856; doi:10.7717/peerj.12313)

A

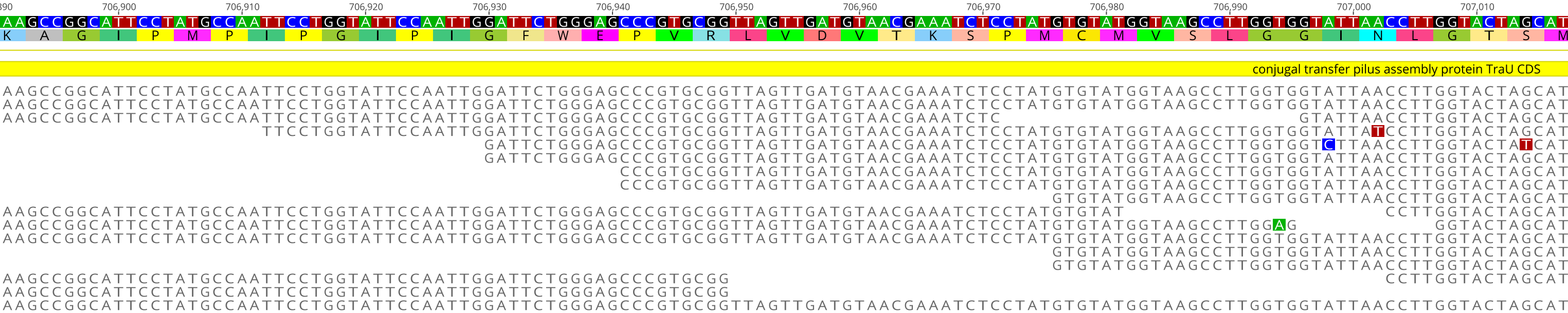

B

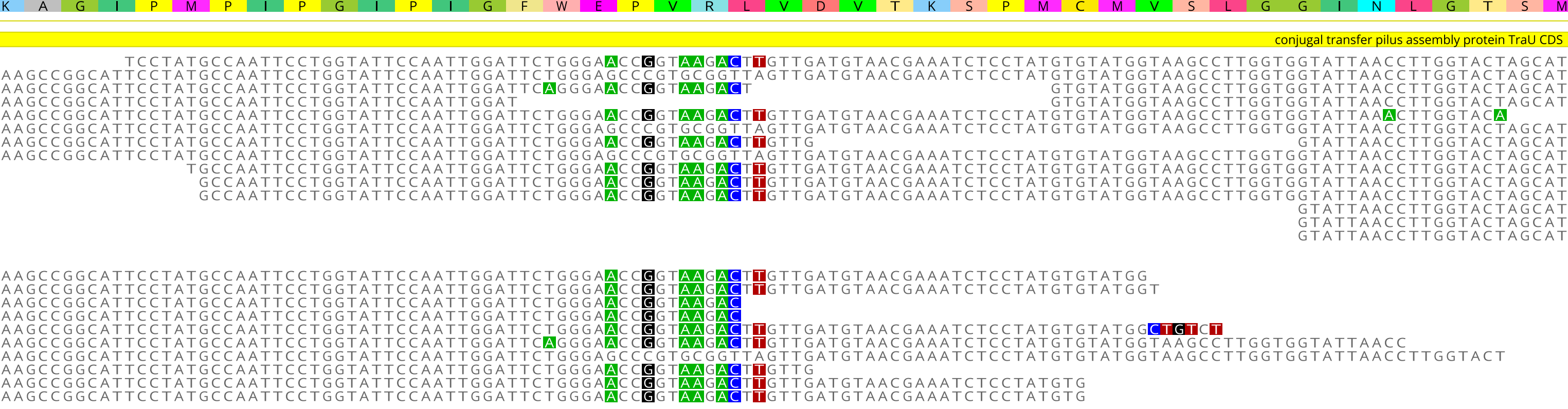

Supplement: Supplemental Information 2 — Illustration of short-read mapping of sequence data to the Rickettsia endosymbiont of Ixodes scapularis str. Wikel genome (NCBI BioProject PRJNA33979. The region spanning nucleotides 706,940 –706,952 (RAGE-Be element) of the symbiont chromosome scaffold CM000770 is identical to the reference genome with regard to (A; top) the NWSE data however contain seven linked polymorphisms in (B; bottom) the PVIL data. All seven variants are synonymous and occur within the conjugal transfer pilus assembly protein TraU gene. [file peerj-09-12313-s002.pdf]
