# Supplementary material for: Comparative hologenomics of two Ixodes scapularis tick populations in New Jersey
Source: PeerJ. 2021 Nov 9;9:e12313. doi: 10.7717/peerj.12313 (PMC8588856; doi:10.7717/peerj.12313)
Supplement: Supplemental Information 3 — Illustration of short-read mapping of sequence data to the Rickettsia endosymbiont of Ixodes scapularis str. Wikel genome (NCBI BioProject PRJNA33979. The polymorphic region spans nucleotides 760,409 –760,434 of the genome scaffold and contains six linked SNVs (hilighted) that are fixed in the (A; top) NWSE data but occur only at low coverage in the (B; bottom) PVIL library. Four polymorphisms are non-synonymous and result in four amino acid changes (illustrated atop each respective alignment) in the conjugative transfer protein TraA gene. [file peerj-09-12313-s003.pdf]

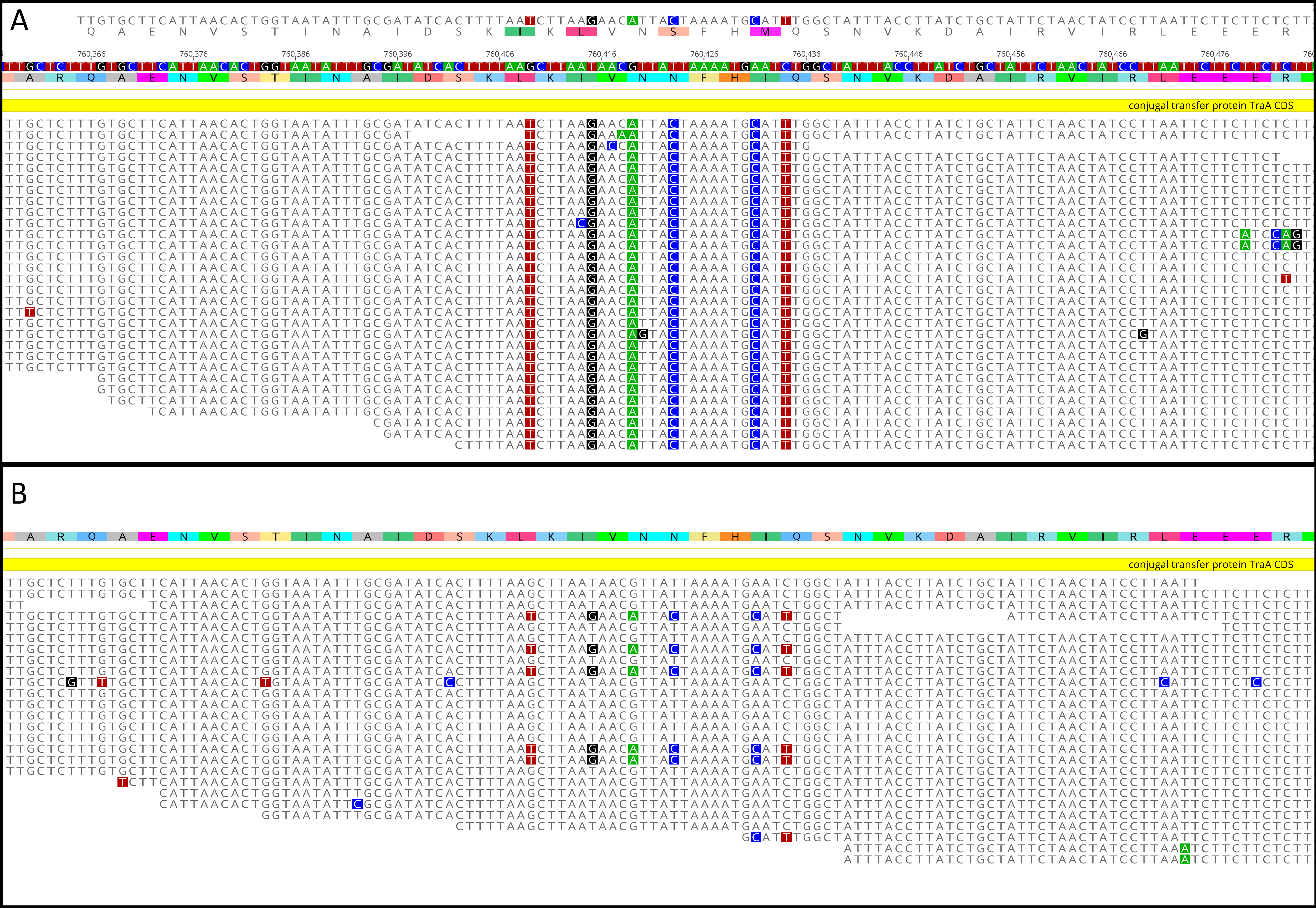
A
760,366
760,376
760,386
760,396
760,406
760,416
760,426
760,436
760,446
760,456
760,466
760,476
conjugal transfer protein TraA CDS
B
conjugal transfer protein TraA CDS
